# Dialkyl Carbamoyl Chloride (DACC)-Impregnated Dressings for the Prevention of Surgical Site Infections: Experience From a Multi-disciplinary Study in India

**DOI:** 10.7759/cureus.72654

**Published:** 2024-10-29

**Authors:** Praharsha Mulpur, Tarun Jayakumar, Parag K Sancheti, Navaladi Shankar, Kushal Hippalgaonkar, A.V. Gurava Reddy

**Affiliations:** 1 Orthopaedics, KIMS-Sunshine Hospitals, Hyderabad, IND; 2 Orthopaedics and Trauma, Sancheti Institution for Orthopaedics and Rehabilitation, Pune, IND; 3 Department of Orthopaedics, Apollo Hospitals, Chennai, IND

**Keywords:** antibiotic resistance, dacc, orthopaedic dressing, surgical site infection, wound dressing

## Abstract

Background

Surgical site infections (SSIs) represent a significant burden in healthcare, commonly occurring after surgical procedures and leading to prolonged recovery times and increased healthcare costs. Traditional antimicrobial dressings pose risks such as antimicrobial resistance. This study aimed to evaluate the safety and clinical efficacy of dialkyl carbamoyl chloride (DACC)-impregnated dressings, which use a purely physical mechanism to prevent bacterial contamination, in patients undergoing orthopaedic or gastrointestinal surgeries.

Methods

This prospective, multicentre observational study was conducted after ethical committee approval across four centres in India, involving 106 patients (71 orthopaedic and 35 gastrointestinal) who received DACC-impregnated dressings. Dressings were applied immediately post-surgery and assessed over 30 days for the incidence of superficial or deep SSI. Additional evaluations included pain measured using visual analogue scale (VAS), dressing adhesion, patient satisfaction, and healthcare provider feedback. Statistical analyses included descriptive statistics and comparisons between time points using the Wilcoxon and Kruskal-Wallis tests, with a significance level set at p<0.05.

Results

Among the 106 patients, two (1.9%) cases of SSI were reported, both in orthopaedic patients. The dressings maintained at least 50% adhesion in 98.1% of cases. Pain levels using VAS averaged 3.9 (SD=2.6) at follow-up one and 2.8 (SD=2.8) at follow-up two. Patient satisfaction was high, with 73.5% reporting improved pain experiences during dressing changes compared to previous dressings. Healthcare professionals rated the handling of dressings as excellent in 89% of cases.

Conclusion

DACC-impregnated dressings demonstrated effectiveness in reducing SSIs in postoperative care for orthopaedic and gastrointestinal surgeries. The dressings were well-tolerated by patients and preferred by healthcare providers due to ease of use and high adherence. These findings support DACC-impregnated dressings as a safe and effective alternative for SSI prevention, particularly beneficial in reducing the risks associated with antimicrobial resistance. Further studies with larger sample sizes and controlled designs are recommended to validate these findings.

## Introduction

Surgical site infections (SSIs) are the most common type of hospital-acquired infections worldwide with reported incidences up to 20% [1,2]. They are defined as infections arising within 30-90 days after an operative procedure and can affect both the surficial and deeper tissues around the incision site [3]. They often delay wound healing, increase the length of hospital stay and require re-intervention and treatment (e.g. antibiotic therapy) [2,4].

Despite the improvements in infection prevention strategies, SSIs still cause significant morbidity, mortality and healthcare economic burden [5,6]. According to the US Centers for Disease Control and Prevention (CDC), SSIs account for 20% of all nosocomial infections and are associated with a two- to 11-fold increased risk of mortality [3].

Morbidity and mortality due to SSI can be prevented with appropriate preoperative, intraoperative and postoperative measures [7,8]. Based on a 2018 Cochrane review, antibiotic prophylaxis reduces SSI risk in certain surgical procedures [4]. However, antibiotic use is associated with antimicrobial resistance (AMR), which in turn can exacerbate infections and their global burden [9]. A systematic review in the Lancet estimated 4.95 million deaths associated with bacterial AMR in 2019, of which 1.27 million were attributable to bacterial AMR. On the contrary, the evidence for other intraoperative interventions (e.g. barriers to prevent bacterial contamination) is poor [4].

In the postoperative setting, antimicrobial dressings represent a successful and cost-effective wound care option. They promote optimal wound healing by protecting the wound from bacterial contamination [10]. In such context, dialkyl carbamoyl chloride (DACC)-coated dressings have recently emerged. Unlike traditional antimicrobial dressings with an active antimicrobial agent (e.g., silver, iodine and polyhexamethylene biguanide) [11], DACC-impregnated dressings have a physical mode of action. They irreversibly bind the hydrophobic extracellular surface of bacteria, promoting the removal of these microorganisms with each dressing change [12].

Their efficacy in both treatment and prevention of infection has been demonstrated in a variety of clinical settings and with different bacterial species with no associated risk of antimicrobial resistance or bacterial endotoxin release [1,12,13].

This prospective, multicentre, observational study evaluated the clinical performance and safety of a DACC-impregnated wound dressing in patients with orthopaedic or gastrointestinal surgery.

## Materials and methods

This prospective, multicentre observational study, was conducted at four independent medical institutes, which included KIMS-Sunshine hospitals-Hyderabad, Asian Institute of Gastroenterology-Hyderabad, Apollo Hospitals-Chennai, and Sancheti Institution for Orthopaedics & Rehabilitation, Pune constituted by three orthopaedic departments and one surgical gastroenterology unit, between June 2021 to February 2022. This study was approved by both the institutional ethical committee (SIEC/2021/449) and the national clinical trials registry (CTRI/2021/07/034509). The study enrolled patients undergoing elective orthopaedic or gastrointestinal surgeries who received the study product, a DACC-impregnated wound dressing (Leukomed® Sorbact®, Gothenburg, Sweden), suitable for dry to low-exuding acute surgical wounds.

Eligible participants included adult patients requiring postoperative dressing for 14 days. Exclusion criteria encompassed prior infections at the target wound site, pre-operative antibiotic use for unrelated conditions (excluding surgical prophylaxis or antibiotics related to the index surgery), drug or substance abuse (including alcohol), immunocompromised status, known sensitivity or allergy to dressing components, involvement in other clinical studies, inability to attend follow-up visits, lack of consent, and pregnancy or breastfeeding.

The primary endpoint was the incidence of superficial or deep SSIs over a 30-day follow-up period post-surgery. Secondary endpoints included evaluations of skin damage after dressing removal, pain levels during dressing removal using visual analogue scale (1-10, with 10 being extreme pain), signs of infection, wound and peri-wound skin condition, dressing adherence, patient satisfaction, and caregiver satisfaction.

Dressing application occurred at the initial visit during in-patient stay, which included an assessment of the peri-wound skin conditions such as reddening or skin damage before the dressing application. Follow-up visits were performed five to seven days (follow-up one), 14 days (follow-up two), and 30 days (final visit or follow-up three) after the dressing application. Follow-up visits included the documentation of variations in concomitant medications (especially antibiotics), dressing changes, assessment of infection signs, skin and wound conditions, evaluation of pain (general wound pain and during dressing change) and dressing adhesion. Patients and healthcare professionals also evaluated the study product. Dressing adhesion was categorized into intervals based on the percentage of area retained: >90%, 75-90%, 50-75%, 0-50%, and 0%. Patient satisfaction with wearing comfort was assessed after follow-up two (Day 14) with options ranging from "very satisfied" to "very dissatisfied." Comparisons to previous dressings were also requested, with ratings of "much better," "somewhat better," "similar," "somewhat worse," or "much worse." Caregiver satisfaction with dressing handling, including use with gloves, was similarly graded using this Likert-scale.

Patients could choose between the following ratings: very satisfied, satisfied, indifferent, unsatisfied, very dissatisfied or had the option not to answer. Additionally, the comparison to formerly received dressings was requested with the rating options: Much better, somewhat better, stayed the same, somewhat worse, much worse or the option not to answer. The wound caregiver satisfaction was assessed by evaluating the general handling and handling with gloves for all visits with the possible answer options: Very satisfied, satisfied, indifferent, unsatisfied, very dissatisfied or the option not to answer. A comparison with other formerly used dressings was queried for rating as much better, somewhat better, stayed the same, somewhat worse, much worse or the option not to answer.

Statistical analysis

Continuous variables were expressed as means with standard deviations and/or median and percentiles, and ranges. Percentages and frequencies were used for nominal and ordinal data. Descriptive statistics of baseline data, treatments, safety as well as primary and secondary endpoints were performed for all subjects presenting at the 14-day study visit. Statistical analyses were performed to analyse changes between timepoints e.g. for SSI rates, skin conditions and wound parameters (absolute and relative frequencies; Kruskal Wallis test and Wilcoxon test, if required), or endpoint analyses (mean and standard deviation/percentiles; parametric: student t-test; non-parametric: Wilcoxon rank sum test). Statistical analyses were done using the SAS® software program (SAS Institute Inc., Cary, NC, USA) with P-values below 0.05 considered statistically significant.

Attendance at follow-up three (Day 30) was not required, as most subjects were anticipated to have achieved sufficient wound healing by that time. Therefore, study completion was defined as the successful completion up to follow-up two (Day 14). The recruitment plan, based on the Clinical Investigation Plan (CIP), targeted 110 subjects to account for a 10% drop-out rate (11 out of 110). Ultimately, 112 subjects were recruited, out of whom six subjects (5.4%) were categorized as drop-outs, leading to a final analysis cohort of 106 subjects.

## Results

Demographics and baseline characteristics

The four study centres recruited 112 patients, following which 106 (94.6%) attended the second follow-up (POD-14) and completed the study. Three subjects also attended the final visit (follow-up three). Demographics and baseline characteristics are displayed in Table 1.

**Table 1:** Demographics and baseline characteristics CV: cardiovascular; SD: standard deviation. Values are n (%) unless otherwise specified.

Characteristics	All patients (N = 106)	
	n (%)	Range
Sex		
1. Male	54 (50.9)	-
2. Female	52 (49.1)	-
Age, mean (SD), years	53.2 (14.4)	18.0–91.0
BMI, mean (SD), kg/m^2^	27.0 (4.1)	20.3–40.9
Smoking status		
1. Current smoker	5 (4.7)	-
2. Former smoker	2 (1.9)	-
Comorbidities		
1. Diabetes mellitus	29 (27.4)	-
2. Cardiovascular disease	15 (14.2)	-
3. Hyperthyroidism	1 (0.9)	-
Family history of disease		
1. Diabetes mellitus	29 (27.4)	-
2. Hypertension/CV disease	27 (25.5)	-
3. Other	15 (14.2)	-
Type of surgery		
1. Orthopedic surgery	71 (67.0)	-
2. Gastrointestinal surgery	35 (33.0)	-
Type of orthopaedic surgery		
1. Knee replacement	39 (36.8)	-
2. Posterior lumbar intervertebral fusion	11 (10.4)	-
3. Other	21 (19.8)	-
Length of hospital stay, mean (SD), days	4.2 (1.6)	2.0–7.0
Wound size, mean (SD), cm	15.5 (7.6)	2.0–35.0
Wound location		
1. Knee	49 (46.2)	-
2. Abdomen	35 (33.0)	-
3. Spine	19 (17.9)	-
4. Other	11 (10.4)	-

The study population included 54 (50.9%) men and 52 (49.1%) women with a mean age of 53.2 years (SD 14.4, range: 18.0 to 91.0 years). Patients had a mean BMI of 27.0 kg/m2 (SD 4.1, range: 20.3 to 40.9). Five (4.7%) patients were current smokers, and two (1.9%) patients were former smokers. Concomitant diseases included diabetes mellitus in 29 (27.4%) and cardiovascular diseases in 15 (14.2%) patients.

Seventy-one patients underwent orthopaedic surgeries, with knee replacement (n = 39) and posterior lumbar intervertebral fusion (n = 11) being the most common indications. Gastrointestinal surgeries were performed on 35 patients. Patients left the hospital after a mean of 4.2 days (SD 1.6, range: 2.0-7.0 days). The mean wound size was 15.5 cm (SD 7.6, range: 2.0-35.0). Wounds were located at the knee (n=49), abdomen (n=35), spine (n=19), wrist (n=7), neck (n=2), elbow (n=1), and hip (n=1). Performance evaluation is shown in Table 2.

**Table 2:** Summary of study results a N=106 unless otherwise specified; b Values are n (%) unless otherwise specified; c Five to seven days after dressing application; d Fourteen days after dressing application; e Thirty days after dressing application; f Total number of dressings evaluated for adhesion; g Only three patients attended the final visit; h The evaluation of dressing handling was performed on 246 dressings. SSI: Surgical site infection, VAS: visual analogue scale

Endpoints^a^	n (%)^b^
Primary endpoint	
1. SSI incidence at follow-up 1^c^	2 (1.4)
Secondary endpoints	
1. SSI incidence	
Follow-up 2^d^	0 (0)
Follow-up 3^e^	0 (0)
2. Dressing adhesion (N=215^f^)	
Dressing adhesion >50%	211 (98.1)
Additional dressing required	210 (97.7)
3. VAS after dressing removal, mean (SD)	
Follow-up 1^c^	3.9 (2.6)
Follow-up 2^d^	2.8 (2.8)
Follow-up 3^e,g^	4.0 (1.7)
4. Patient pain experience vs. other dressings	
Much better	17 (16.0)
Somewhat better	61 (57.5)
Similar	24 (22.6)
5. Overall patient wearing comfort	
Very satisfied	46 (43.4)
Satisfied	41 (38.7)
6. Patient wearing comfort vs. other dressings	
Much better	58 (54.7)
Somewhat better	26 (24.5)
7. Overall healthcare professional satisfaction with handling (N=246^h^)	
Excellent	219 (89.0)
Above average	26 (10.6)
Average	1 (0.4)
8. Healthcare professional satisfaction with handling with gloves	
Excellent	222 (90.2)
Above average	24 (9.8)

Incidence of surgical site infection

Signs of a suspected infection were documented in four subjects (3.8%) at the first follow-up visit. No further infections were documented during the second and final follow-up visit. Out of four patients with infection signs, SSI was only confirmed for two patients, resulting in an SSI rate of 1.9%. Both SSIs were described as superficial, whereas no details were provided for the other two patients with infection signs. Both SSIs occurred in the group of orthopaedic surgery.

Dressing adhesion

**Figure 1:**
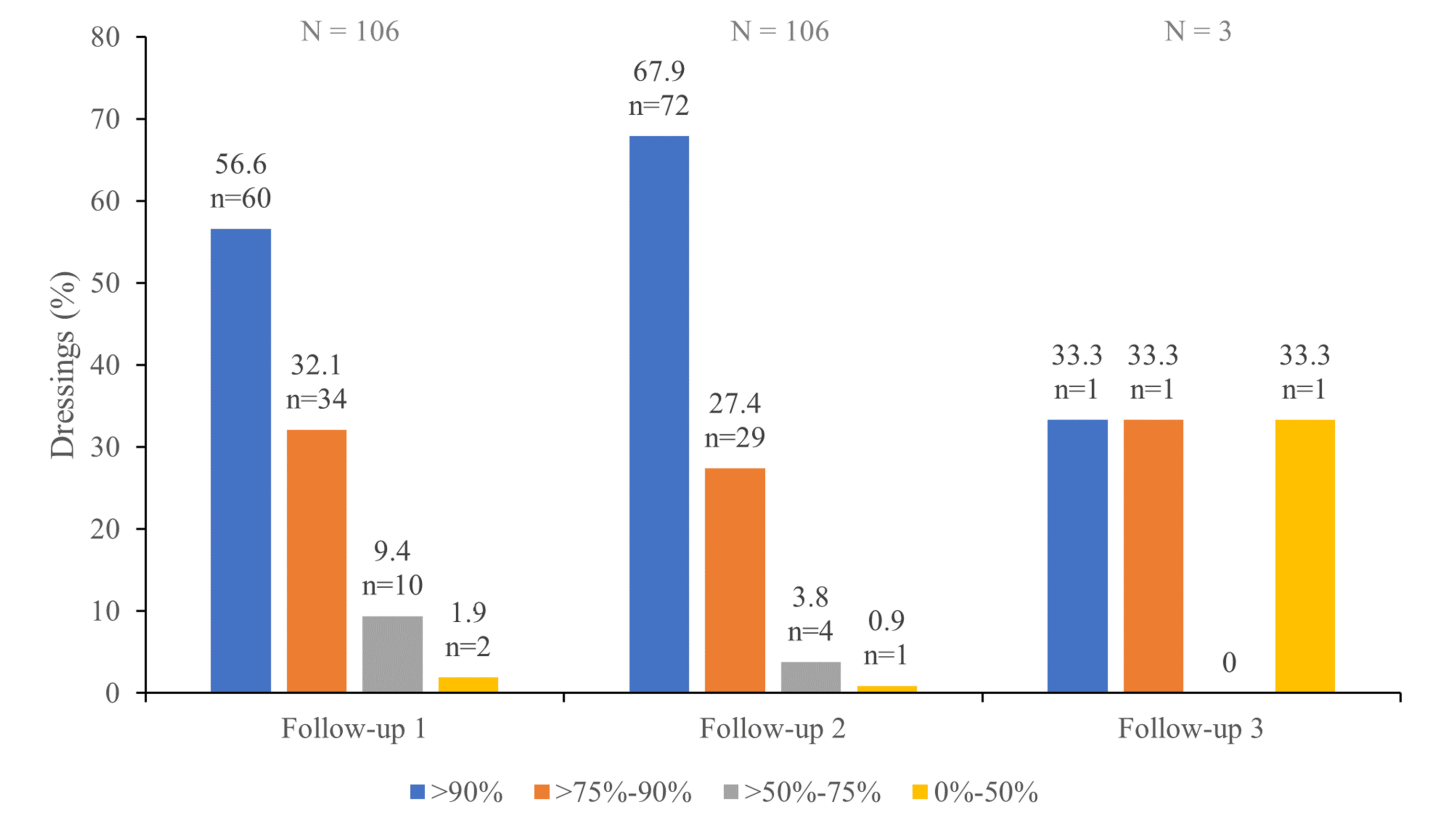
Dressing Adhesion

The evaluation of dressing adhesion was performed in 215 dressings. The dressings stayed in place with at least 50% of its adhesive area in all but four cases (Figure 1).

Dressing changes were only planned for the respective follow-up visits. Additional dressing changes were required in five cases (2.3%). No additional changes were required in the remaining 210 cases (97.7%).

Healthcare professional and patient satisfaction

**Figure 2:**
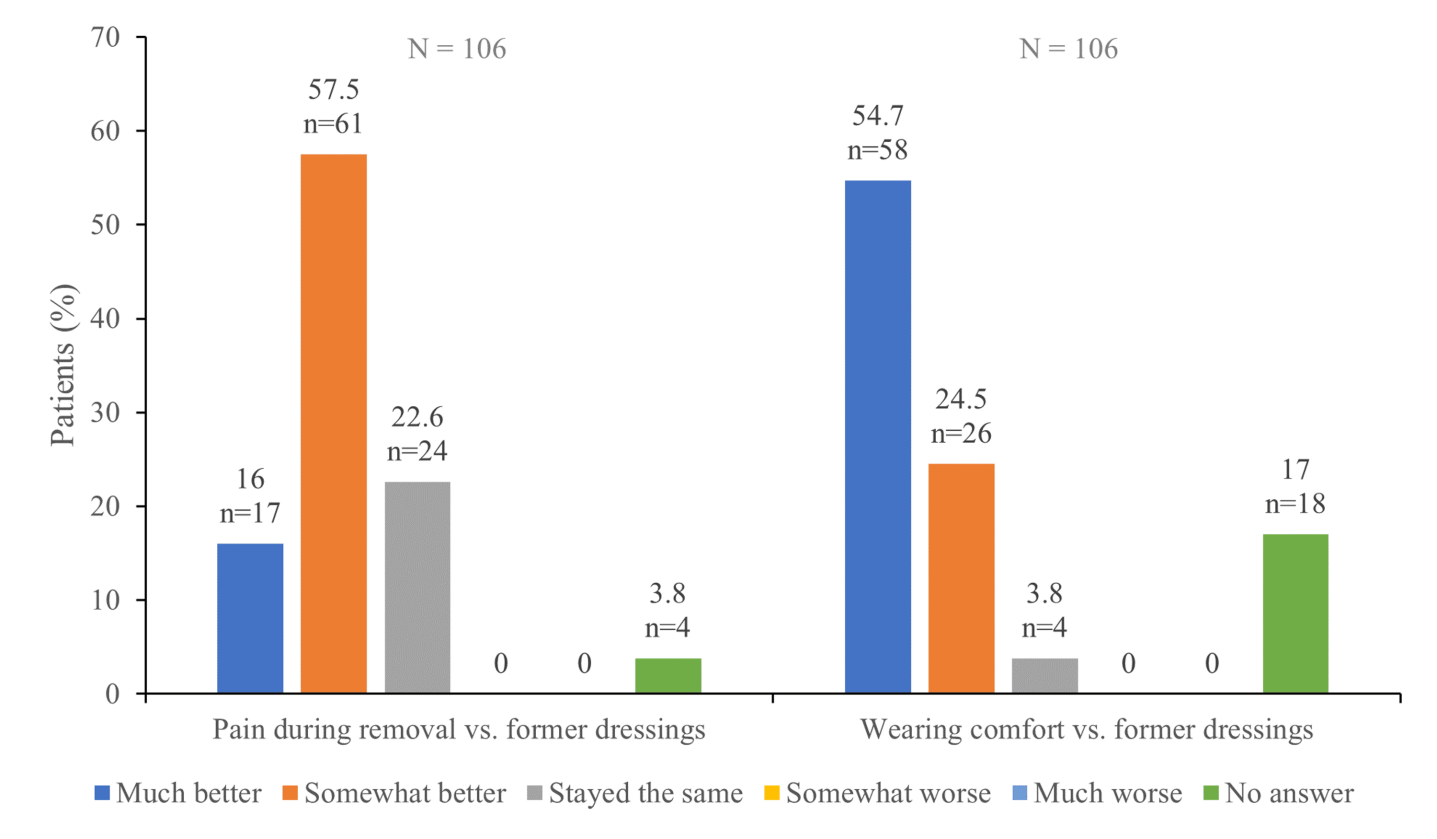
Patient satisfaction with dialkyl carbamoyl chloride (DACC)-impregnated dressings compared with other dressings

Pain assessment with the VAS was performed after each dressing change. The mean VAS after dressing removal was 3.9 (SD 2.6) at the first follow-up visit and 2.8 (SD 2.8) at the second follow-up visit. Only three subjects attended the final visit and reported a VAS scale of 4.0 (SD 1.7). The pain experience during dressing removal was defined as much better or somewhat better than the removal of other dressings in 17 (16.0%) and 61 (57.5%) patients, respectively. Twenty-four (22.6%) patients reported a similar pain than that experienced with past wound dressings (Figure 2). Forty-six (43.4%) and 41 (38.7%) patients were very satisfied or satisfied with the wearing comfort. Compared to other products received for former treatments, the wearing comfort of the study device was evaluated much better in 58 (54.7%) or somewhat better in 26 (24.5%) of patients (Figure 2).

**Figure 3:**
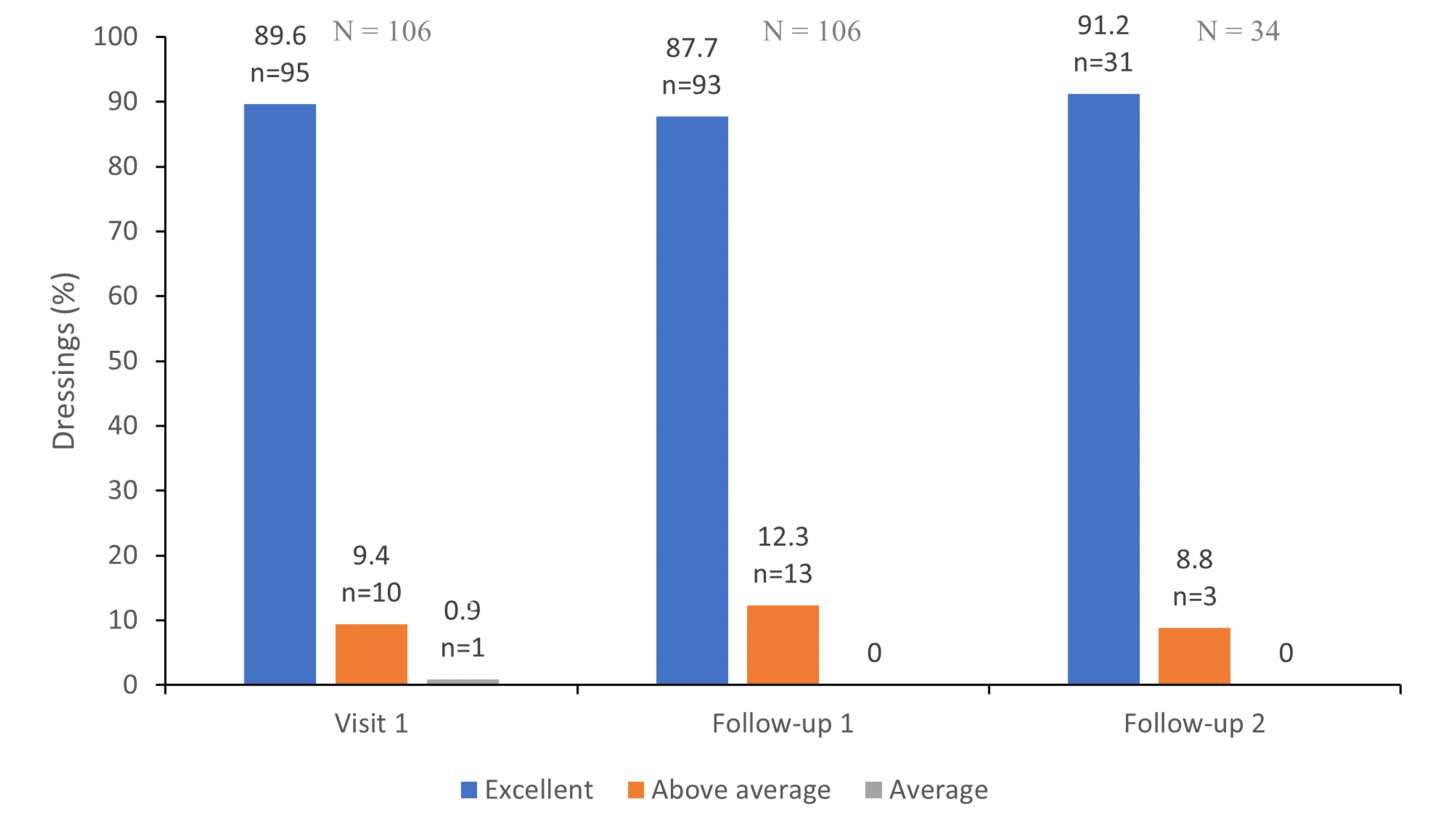
Healthcare professional satisfaction with the dressing application

Healthcare professionals were asked to judge the handling of the study device in general and the handling with gloves. Out of the 246 dressing applications, 245 (99.6%) were categorised as above average or excellent; the dressing application was defined as average only in one case (Figure 3). None of the healthcare professionals described the handling as below average or very poor. The handling with gloves was positively described for all applications for follow-up visits one to three.

Safety evaluation

Safety parameters included clinically relevant skin reactions or changes in wound conditions, signs of infections (other than SSI), readmission to hospital and time of hospitalisation, adverse device effects (ADE), serious adverse events (SAE), serious adverse device effects (SADE) and device deficiency (DD).

Clinically relevant skin reactions and skin damages

Skin damage occurred in 0.5% of dressing changes, described as tension injury or blister. No other skin tears, skin stripping or other skin reactions (e.g. irritant contact dermatitis, allergic dermatitis, maceration, or folliculitis) were detected.

Adverse events

No SADE or device deficiencies were reported. One patient experienced an SAE at 14 days. A blood clot at the wound required wound debridement and hospitalisation. The event was not related to the device.

## Discussion

This study evaluated the clinical performance and safety of a DACC-impregnated wound dressing in patients undergoing gastrointestinal or orthopaedic surgeries. An overall SSI rate of 1.9% (two out of 106 patients) was found; both infections occurred in the orthopaedic trauma surgery group, whereas no signs of infections were documented in the gastrointestinal surgery patients. Therefore, the incidence of SSI was 2.8% if only the orthopaedic surgery group was taken into consideration.

It is well known that the type of surgery affects the incidence of SSI [2,14]. As opposed to our study, Indian studies report a higher incidence of SSIs for gastrointestinal surgery (6.7-17.2%) than orthopaedic trauma surgery (4-5.5%) [15-21]. The low SSI rate observed with our DACC-impregnated dressing aligns with existing global evidence. A randomised controlled trial in 543 patients undergoing caesarean section reported an SSI rate of 1.8% at 14 days post-surgery with DACC-impregnated wound dressings compared with 5.2% when standard surgical dressings were used [22]. Furthermore, DACC-impregnated dressings were evaluated in a non-randomised comparative study after nonimplant vascular surgery; a significantly lower SSI rate was observed five days post-surgery compared with conventional dressings [23]. Lastly, a pilot feasibility study comparing DACC-impregnated dressing with standard of care for the prevention of SSI in vascular surgery patients showed a non-significant relative risk reduction of 36.9% in the DACC-impregnated wound dressing arm [24]. All studies in vascular surgery patients and caesarean section women found a lower rate of SSI with DACC-impregnated dressings compared with standard surgical dressings. However, the results were not always statistically significant.

As skin adhesion is an important performance parameter for wound dressing, our analysis also included the evaluation of the dressing skin adhesion and the frequency of dressing change. Skin adhesion assessment revealed that most dressings stayed in place with at least 50% of their adhesive area [2,4,14]. Regarding dressing change frequency, dressing changes were planned during the visits (seven to nine days between visit one and follow-up one, seven days between follow-up one and two, and 16 days between follow-up two and three). No additional changes were required in 97.7% of cases. Based on our data, DACC-impregnated dressings provide reliable wound covering and adequate skin adhesion, which are, indeed, key factors to allow wound healing without unnecessary contamination and promote adequate tissue formation and skin recovery [1,25,26]. Our device offers a similar/more convenient dressing change frequency when compared with alternative orthopaedic surgical dressings (≥seven days compared with two to 10 days; the change frequency for the majority of alternative orthopaedic surgical dressings reviewed by Tantillo et al. is two days).

With respect to patients and healthcare professionals' satisfaction with the dressing, the level of satisfaction in our study was high for all the parameters evaluated (i.e. pain experienced during dressing removal, wearing comfort and handling of the dressing). Similar findings were reported in a study by Pickles et al. where most healthcare professionals were satisfied, very satisfied or extremely satisfied with the application of the DACC-impregnated dressing [27].

This study also demonstrated a beneficial safety profile for our DACC-impregnated dressing. Only one (serious) adverse event (i.e. bleeding and clotting requiring medical intervention) was reported overall and was not related to the investigational device. Indeed, unexpected bleeding and clotting are common postoperative complications after orthopaedic surgery; they are related to the surgical procedure rather than the postoperative dressing [28]. Furthermore, although skin injury is known to be associated with application and removal of surgical wound dressing with a different extent based on the type of dressing [26,29], our study reported no skin reaction due to mechanical stress of the skin. Similarly, no skin reactions due to sensitivity or allergy to product components were observed.

Based on the positive results of our study, DACC-impregnated dressings represent a safe and effective option to prevent SSIs in a variety of surgical procedures. Given the increasing global burden of AMR associated with the abuse or misuse of antibiotics (i.e. 4.95 million AMR-associated deaths registered in 2019 and 10 million estimated by 2050) [30,31], our device is particularly promising as it does not involve the use of antimicrobial agents. The consideration of the health economic impact of DACC-impregnated dressings also supports the use of our DACC-impregnated device. A cost analysis based on the randomised controlled trial in women undergoing caesarean section by Stanirowski et al. revealed a cost reduction of 57.6% when DACC-impregnated dressings are used instead of the standard of care [22].

Nevertheless, our results should be interpreted in light of some limitations. Firstly, our study was an uncontrolled observational study with no comparator. Therefore, the resulting SSI rates can only be compared with the literature and methodological differences between our study and those published in the literature must be taken into consideration when comparing the results. Moreover, not all subjects completed the device performance evaluation at follow-up two.

Although our study demonstrated a beneficial, well-accepted and safe use of DACC-impregnated dressings in numerous surgical settings, rare ADE might not have been captured cause of the small number of patients. Larger post-marketing clinical evaluation studies or the analysis of data from hospital registries during continuous post-marketing surveillance might reveal further insights. Furthermore, the benefit of DACC-impregnated dressings in reducing SSI rate is highly dependent on the type and duration of surgery as well as the patient’s overall physical health, which are aspects that have not been thoroughly investigated in our study. Therefore, future controlled clinical studies with subgroup analysis for comorbidities, family history of disease and type/duration of surgery are warranted to gather detailed insights on SSI reduction.

## Conclusions

Based on the results of this post-market clinical evaluation study, DACC-impregnated dressings successfully maintained surgical site infection prevention and is a safe postoperative dressing after orthopaedic and gastrointestinal surgery with high healthcare professional and patient acceptance. Furthermore, it has the potential to improve patient’s quality of life by improving wearing comfort and offering optimal wound healing with a minor risk of infections and no risk of antimicrobial resistance. The need for future controlled clinical studies or analysis of large registries is highlighted to better characterise the clinical performance and safety of DACC-impregnated dressings in specific patient populations and surgical settings.
